# Probing information routing mechanisms by precisely-timed electrical stimulation pulses: a modelling study

**DOI:** 10.1186/1471-2202-16-S1-P70

**Published:** 2015-12-18

**Authors:** Dmitriy Lisitsyn, Daniel Harnack, Udo Ernst

**Affiliations:** 1 Institute for Theoretical Physics, Computational Neuroscience Lab, University of Bremen, Bremen, D-28159, Germany

## 

In dependence on the behavioral context, sensory signals are selectively routed throughout the brain. Particularly, in area V4 of the visual cortex, neurons with multiple stimuli in their receptive fields respond as if just the attended stimulus was present [1]. The mechanisms for selective information processing are still debated, but gamma-band synchronization between brain areas such as V1 and V4 emerged as a promising candidate, proposing that information is routed between populations with a synchronized in-phase relationship and blocked when gamma oscillations are in an anti-phase or random relationship [2] (communication-through-coherence, CTC).

The causality of this hypothesis has proven hard to establish, but cortical stimulation gives us the tools to investigate it. By utilizing controlled perturbations, we could shift neural populations into desired phase relationships and quantify the effect on visual information gating. As a precursor to animal experiments, we investigate this paradigm by using neural models to predict the effect of stimulation on gamma oscillations, studying the feasibility of different synchronization scenarios and their robustness against noise.

Without stimulation, the troughs of the unpulsed V4 LFP (in grey) coincide with the peaks of the V1 population B (in red), hence suppressing the incoming information from B while "attending" to the signal carried by A. However, a pulse applied at the proper phase of the V4 LFP (marked in green), results in a phase shift of the LFP (in black). The troughs of the new LFP now coincide with V1 population A, thus effectively changing which input signal is gated/attended.

**Figure 1:**
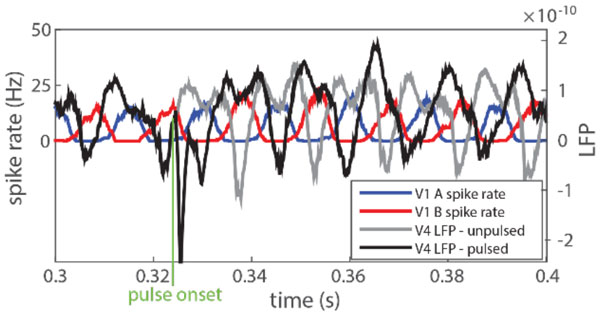
**LFP and spike data of the model**. Without stimulation, the troughs of the unpulsed V4 LFP (in grey) coincide with the peaks of the V1 population B (in red), hence suppressing the incoming information from B while "attending" to the signal carried by A. However, a pulse applied at the proper phase of the V4 LFP (marked in green), results in a phase shift of the LFP (in black). The troughs of the new LFP now coincide with V1 population A, thus effectively changing which input signal is gated/attended.

First, we explored the effect of precisely-timed electric pulses on different oscillation mechanisms proposed for generating γ-rhythms, specifically ING and PING [3], for which we obtained phase-response curves (PRC) under multiple stimulation regimes. Using these oscillation mechanisms, we constructed a simple network representing two V1 populations A and B, and one V4 population. When simultaneously driven by two input stimuli, the two V1 populations would oscillate in anti-phase, while the V4 population would synchronize with V1 populations A and B in an alternate fashion (bistable dynamics). Based on the computed PRC's, a stimulation paradigm was developed which monitored the relative phase relations of the populations online, allowing us to pulse V4 at the right time to successfully control which V1 population the V4 population would synchronize with, thus enhancing the information transfer between these two modules (see Figure 1).

The success of stimulation strongly depends on the noise level and the resulting reliability of Gamma oscillations. At a critical point, shifting phases gradually is no longer feasible and a stronger phase-reset pulse has to be used instead. In addition, a careful monitoring of on-going activity is essential since phase control is most efficient in periods where the observed Gamma amplitude is high.
